# 3-(1*H*-Imidazol-1-yl)-1-phenyl­propan-1-ol

**DOI:** 10.1107/S1600536812004254

**Published:** 2012-02-10

**Authors:** Hoong-Kun Fun, Ching Kheng Quah, Mohamed I. Attia, Hatem A. Abdel-Aziz, Khalid A. Al-Rashood

**Affiliations:** a X-ray Crystallography Unit, School of Physics, Universiti Sains Malaysia, 11800 USM, Penang, Malaysia; b Department of Pharmaceutical Chemistry, College of Pharmacy, King Saud University, Riyadh 11451, Saudi Arabia

## Abstract

In the title compound, C_12_H_14_N_2_O, the imidazole ring forms a dihedral angle of 66.73 (5)° with the phenyl ring. In the crystal, mol­ecules are linked *via* O—H⋯N and C—H⋯O hydrogen bonds into sheets lying parallel to (100). The crystal structure is further consolidated by C—H⋯π inter­actions.

## Related literature

For general background to and the pharmacological activities of the title compound, see: Latge (1999 ▶); Steenbergn & Casadevall (2000 ▶); Pacetti & Gelone (2003 ▶); Spellberg *et al.* (2006 ▶). For the preparation of the title compound, see: Aboul-Enein *et al.* (2011 ▶). For the stability of the temperature controller used for the data collection, see: Cosier & Glazer (1986 ▶).
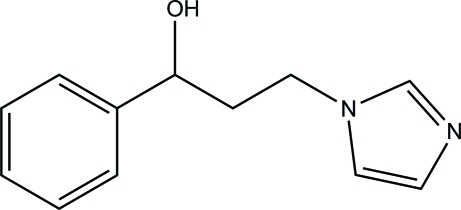


## Experimental

### 

#### Crystal data

C_12_H_14_N_2_O
*M* *_r_* = 202.25
Monoclinic, 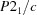

*a* = 9.0352 (5) Å
*b* = 11.8521 (7) Å
*c* = 10.3462 (6) Å
β = 109.688 (1)°
*V* = 1043.17 (10) Å^3^
*Z* = 4
Mo *K*α radiation
μ = 0.08 mm^−1^
*T* = 100 K
0.34 × 0.26 × 0.19 mm

#### Data collection

Bruker SMART APEXII DUO CCD diffractometer
Absorption correction: multi-scan (*SADABS*; Bruker, 2009 ▶) *T*_min_ = 0.972, *T*_max_ = 0.985
14426 measured reflections
3777 independent reflections
3245 reflections with *I* > 2σ(*I*)
*R*_int_ = 0.022

#### Refinement

*R*[*F* ^2^ > 2σ(*F* ^2^)] = 0.043
*wR*(*F* ^2^) = 0.125
*S* = 1.05
3777 reflections
140 parameters
H atoms treated by a mixture of independent and constrained refinement
Δρ_max_ = 0.67 e Å^−3^
Δρ_min_ = −0.26 e Å^−3^

### 

Data collection: *APEX2* (Bruker, 2009 ▶); cell refinement: *SAINT* (Bruker, 2009 ▶); data reduction: *SAINT*; program(s) used to solve structure: *SHELXTL* (Sheldrick, 2008 ▶); program(s) used to refine structure: *SHELXTL*; molecular graphics: *SHELXTL*; software used to prepare material for publication: *SHELXTL* and *PLATON* (Spek, 2009 ▶).

## Supplementary Material

Crystal structure: contains datablock(s) global, I. DOI: 10.1107/S1600536812004254/hb6620sup1.cif

Structure factors: contains datablock(s) I. DOI: 10.1107/S1600536812004254/hb6620Isup2.hkl

Supplementary material file. DOI: 10.1107/S1600536812004254/hb6620Isup3.cml

Additional supplementary materials:  crystallographic information; 3D view; checkCIF report

## Figures and Tables

**Table 1:** Hydrogen-bond geometry (Å, °) *Cg*1 is the centroid of the C1–C6 phenyl ring.

*D*—H⋯*A*	*D*—H	H⋯*A*	*D*⋯*A*	*D*—H⋯*A*
O1—H1O1⋯N2^i^	0.964 (19)	1.89 (2)	2.8432 (12)	171.7 (17)
C1—H1*B*⋯O1^ii^	0.95	2.48	3.4188 (12)	172
C10—H10*A*⋯*Cg*1^iii^	0.95	2.68	3.4778 (12)	142
C12—H12*A*⋯*Cg*1^iv^	0.95	2.69	3.5373 (11)	149

Symmetry codes: (i) 
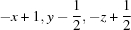
; (ii) 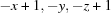; (iii) 
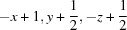
; (iv) 
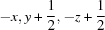
.

**Fig. 1.**
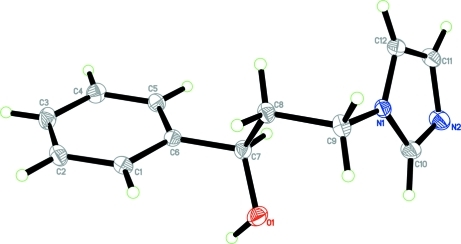
The molecular structure of the title compound showing 50% probability displacement ellipsoids for non-H atoms.

**Fig. 2.**
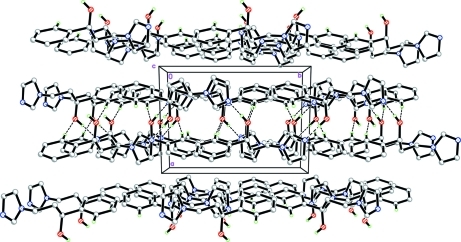
The crystal structure of the title compound, viewed along the c axis. H atoms not involved in hydrogen bonds (dashed lines) have been omitted for clarity.

## supplementary crystallographic information

### Comment

Over the past three decades, the incidence of both community-acquired and nosocomial invasive fungal infections has increased substantially. Clinically, candidosis, aspergillosis and cryptococcosis have been identified as three major opportunistic pathogens in the etiology of fungal infections in immune compromised patients (Latge, 1999; Steenbergn & Casadevall, 2000). Candida albicans accounts for the majority of invasive and superficial Candida infections (Pacetti & Gelone, 2003; Spellberg *et al.*, 2006). Accordingly, the need for new antifungal drugs has prompted intensive research worldwide. The azole antifungal drugs constitute one of the major classes which are characterized by having the azole pharmacophoric moiety embedded in their structures. The title molecule exhibited anti-Candida activity (MIC = 10 µg/ml) and can serve as a prototypic molecule for subsequent molecular modifications.

In the title compound, Fig. 1, the imidazole ring (N1/N2/C10–C12, maximum deviation of 0.002 (1) Å at atoms N1 and C12) forms a dihedral angle of 66.73 (5)° with the phenyl ring (C1–C6).

In the crystal structure, Fig. 2, molecules are linked *via* intermolecular O1—H1O1···N2 and C1—H1B···O1 hydrogen bonds (Table 1) into two-dimensional networks parallel to (100). The crystal structure is further consolidated by C10—H10A···Cg1^iii^ and C12—H12A···Cg1^iv^ (Table 1) interactions, where Cg1 is the centroid of C1–C6 phenyl ring.

### Experimental

NaBH_4_ (4.9 g, 0.13 mol) was added portion-wise to an ice cooled, stirred solution of 3-(1*H*-imidazol-1-yl)-1-phenylpropan-1-one (8.6 g, 0.043 mol) (Aboul-Enein *et al.*, 2011) in methanol (100 ml). The mixture was stirred overnight at ambient temperature followed by evaporation of methanol under vacuum. The residue was dissolved in ethyl acetate (150 ml) and washed with water (3 × 50 ml). The organic layer was separated, dried (Na_2_SO_4_) and evaporated under reduced pressure. The residue was recrystallized from ethanol to give the title compound as colourless blocks. M.p. = 380–382 K.

### Refinement

Atom H1O1 was located in a difference Fourier map and refined freely with O1—H1O1 = 0.97 (2) Å. The remaining H atoms were positioned geometrically and refined using a riding model with C—H = 0.95 or 0.99 Å and *U*_iso_(H) = 1.2*U*_eq_(C).

### Crystal data

**Table:**

C_12_H_14_N_2_O	*F*(000) = 432
*M**_r_* = 202.25	*D*_x_ = 1.288 Mg m^−^^3^
Monoclinic, *P*2_1_/*c*	Mo *K*α radiation, λ = 0.71073 Å
Hall symbol: -P 2ybc	Cell parameters from 6547 reflections
*a* = 9.0352 (5) Å	θ = 3.0–32.6°
*b* = 11.8521 (7) Å	µ = 0.08 mm^−^^1^
*c* = 10.3462 (6) Å	*T* = 100 K
β = 109.688 (1)°	Block, colourless
*V* = 1043.17 (10) Å^3^	0.34 × 0.26 × 0.19 mm
*Z* = 4	

### Data collection

**Table:**

Bruker SMART APEXII DUO CCD diffractometer	3777 independent reflections
Radiation source: fine-focus sealed tube	3245 reflections with *I* > 2σ(*I*)
Graphite monochromator	*R*_int_ = 0.022
φ and ω scans	θ_max_ = 32.6°, θ_min_ = 2.7°
Absorption correction: multi-scan (*SADABS*; Bruker, 2009)	*h* = −13→12
*T*_min_ = 0.972, *T*_max_ = 0.985	*k* = −17→17
14426 measured reflections	*l* = −14→15

### Refinement

**Table:**

Refinement on *F*^2^	Primary atom site location: structure-invariant direct methods
Least-squares matrix: full	Secondary atom site location: difference Fourier map
*R*[*F*^2^ > 2σ(*F*^2^)] = 0.043	Hydrogen site location: inferred from neighbouring sites
*wR*(*F*^2^) = 0.125	H atoms treated by a mixture of independent and constrained refinement
*S* = 1.05	*w* = 1/[σ^2^(*F*_o_^2^) + (0.0644*P*)^2^ + 0.3608*P*] where *P* = (*F*_o_^2^ + 2*F*_c_^2^)/3
3777 reflections	(Δ/σ)_max_ = 0.001
140 parameters	Δρ_max_ = 0.67 e Å^−^^3^
0 restraints	Δρ_min_ = −0.26 e Å^−^^3^

### Special details

**Table:**

Experimental. The crystal was placed in the cold stream of an Oxford Cryosystems Cobra open-flow nitrogen cryostat (Cosier & Glazer, 1986) operating at 100.0 (1) K.
Geometry. All esds (except the esd in the dihedral angle between two l.s. planes) are estimated using the full covariance matrix. The cell esds are taken into account individually in the estimation of esds in distances, angles and torsion angles; correlations between esds in cell parameters are only used when they are defined by crystal symmetry. An approximate (isotropic) treatment of cell esds is used for estimating esds involving l.s. planes.
Refinement. Refinement of F^2^ against ALL reflections. The weighted R-factor wR and goodness of fit S are based on F^2^, conventional R-factors R are based on F, with F set to zero for negative F^2^. The threshold expression of F^2^ > 2sigma(F^2^) is used only for calculating R-factors(gt) etc. and is not relevant to the choice of reflections for refinement. R-factors based on F^2^ are statistically about twice as large as those based on F, and R-factors based on ALL data will be even larger.

### Fractional atomic coordinates and isotropic or equivalent isotropic displacement parameters (Å^2^)

**Table:**

	*x*	*y*	*z*	*U*_iso_*/*U*_eq_	
O1	0.48286 (8)	0.08562 (6)	0.36457 (8)	0.02067 (16)	
N1	0.25481 (8)	0.31589 (7)	0.35682 (8)	0.01420 (15)	
N2	0.32358 (10)	0.45590 (7)	0.24613 (9)	0.02066 (17)	
C1	0.31643 (10)	−0.13411 (8)	0.29554 (9)	0.01560 (16)	
H1B	0.3689	−0.1285	0.3918	0.019*	
C2	0.27877 (11)	−0.23971 (8)	0.23471 (10)	0.01808 (18)	
H2B	0.3073	−0.3059	0.2892	0.022*	
C3	0.19909 (12)	−0.24865 (8)	0.09354 (10)	0.01975 (18)	
H3A	0.1735	−0.3208	0.0519	0.024*	
C4	0.15757 (11)	−0.15146 (9)	0.01470 (9)	0.01935 (18)	
H4A	0.1013	−0.1570	−0.0809	0.023*	
C5	0.19809 (11)	−0.04577 (8)	0.07534 (9)	0.01624 (17)	
H5A	0.1713	0.0203	0.0203	0.019*	
C6	0.27760 (10)	−0.03610 (8)	0.21592 (9)	0.01380 (16)	
C7	0.32242 (10)	0.07925 (8)	0.28116 (9)	0.01549 (16)	
H7A	0.3017	0.1370	0.2066	0.019*	
C8	0.22380 (10)	0.10796 (8)	0.37099 (9)	0.01587 (16)	
H8A	0.1118	0.1116	0.3124	0.019*	
H8B	0.2350	0.0464	0.4383	0.019*	
C9	0.26905 (11)	0.21929 (8)	0.44836 (9)	0.01656 (17)	
H9A	0.2007	0.2317	0.5044	0.020*	
H9B	0.3789	0.2142	0.5117	0.020*	
C10	0.37482 (11)	0.37408 (8)	0.33713 (10)	0.01860 (18)	
H10A	0.4828	0.3579	0.3833	0.022*	
C11	0.16177 (11)	0.44949 (8)	0.20509 (10)	0.01811 (18)	
H11A	0.0914	0.4980	0.1397	0.022*	
C12	0.11734 (10)	0.36343 (8)	0.27205 (9)	0.01602 (17)	
H12A	0.0131	0.3411	0.2621	0.019*	
H1O1	0.543 (2)	0.0448 (17)	0.3186 (19)	0.048 (5)*	

### Atomic displacement parameters (Å^2^)

**Table:**

	*U*^11^	*U*^22^	*U*^33^	*U*^12^	*U*^13^	*U*^23^
O1	0.0152 (3)	0.0220 (4)	0.0251 (3)	−0.0023 (2)	0.0071 (3)	−0.0072 (3)
N1	0.0142 (3)	0.0116 (3)	0.0184 (3)	0.0000 (2)	0.0076 (3)	−0.0010 (2)
N2	0.0182 (3)	0.0179 (4)	0.0290 (4)	0.0007 (3)	0.0120 (3)	0.0036 (3)
C1	0.0146 (3)	0.0167 (4)	0.0159 (3)	0.0024 (3)	0.0057 (3)	0.0012 (3)
C2	0.0204 (4)	0.0137 (4)	0.0232 (4)	0.0028 (3)	0.0114 (3)	0.0018 (3)
C3	0.0227 (4)	0.0154 (4)	0.0244 (4)	−0.0015 (3)	0.0121 (3)	−0.0055 (3)
C4	0.0207 (4)	0.0212 (4)	0.0160 (4)	−0.0009 (3)	0.0060 (3)	−0.0035 (3)
C5	0.0174 (4)	0.0158 (4)	0.0159 (4)	0.0010 (3)	0.0062 (3)	0.0007 (3)
C6	0.0125 (3)	0.0141 (4)	0.0160 (3)	−0.0003 (3)	0.0063 (3)	−0.0015 (3)
C7	0.0155 (3)	0.0142 (4)	0.0180 (4)	−0.0009 (3)	0.0072 (3)	−0.0011 (3)
C8	0.0176 (4)	0.0127 (4)	0.0205 (4)	−0.0004 (3)	0.0106 (3)	−0.0001 (3)
C9	0.0207 (4)	0.0135 (4)	0.0176 (4)	0.0012 (3)	0.0091 (3)	0.0003 (3)
C10	0.0141 (4)	0.0174 (4)	0.0262 (4)	−0.0003 (3)	0.0092 (3)	0.0014 (3)
C11	0.0176 (4)	0.0168 (4)	0.0219 (4)	0.0032 (3)	0.0092 (3)	0.0020 (3)
C12	0.0136 (3)	0.0160 (4)	0.0195 (4)	0.0003 (3)	0.0069 (3)	−0.0012 (3)

### Geometric parameters (Å, º)

**Table:**

O1—C7	1.4173 (11)	C4—H4A	0.9500
O1—H1O1	0.97 (2)	C5—C6	1.3931 (12)
N1—C10	1.3571 (11)	C5—H5A	0.9500
N1—C12	1.3767 (11)	C6—C7	1.5180 (12)
N1—C9	1.4636 (12)	C7—C8	1.5269 (12)
N2—C10	1.3218 (13)	C7—H7A	1.0000
N2—C11	1.3802 (12)	C8—C9	1.5260 (13)
C1—C2	1.3907 (13)	C8—H8A	0.9900
C1—C6	1.3986 (12)	C8—H8B	0.9900
C1—H1B	0.9500	C9—H9A	0.9900
C2—C3	1.3979 (14)	C9—H9B	0.9900
C2—H2B	0.9500	C10—H10A	0.9500
C3—C4	1.3881 (14)	C11—C12	1.3671 (13)
C3—H3A	0.9500	C11—H11A	0.9500
C4—C5	1.3936 (13)	C12—H12A	0.9500
			
C7—O1—H1O1	107.8 (11)	C6—C7—C8	110.39 (7)
C10—N1—C12	106.94 (8)	O1—C7—H7A	108.7
C10—N1—C9	126.45 (8)	C6—C7—H7A	108.7
C12—N1—C9	126.60 (7)	C8—C7—H7A	108.7
C10—N2—C11	105.03 (8)	C9—C8—C7	113.82 (7)
C2—C1—C6	120.42 (8)	C9—C8—H8A	108.8
C2—C1—H1B	119.8	C7—C8—H8A	108.8
C6—C1—H1B	119.8	C9—C8—H8B	108.8
C1—C2—C3	120.16 (9)	C7—C8—H8B	108.8
C1—C2—H2B	119.9	H8A—C8—H8B	107.7
C3—C2—H2B	119.9	N1—C9—C8	112.79 (7)
C4—C3—C2	119.55 (9)	N1—C9—H9A	109.0
C4—C3—H3A	120.2	C8—C9—H9A	109.0
C2—C3—H3A	120.2	N1—C9—H9B	109.0
C3—C4—C5	120.22 (8)	C8—C9—H9B	109.0
C3—C4—H4A	119.9	H9A—C9—H9B	107.8
C5—C4—H4A	119.9	N2—C10—N1	111.96 (8)
C6—C5—C4	120.59 (8)	N2—C10—H10A	124.0
C6—C5—H5A	119.7	N1—C10—H10A	124.0
C4—C5—H5A	119.7	C12—C11—N2	110.28 (8)
C5—C6—C1	119.04 (8)	C12—C11—H11A	124.9
C5—C6—C7	120.32 (8)	N2—C11—H11A	124.9
C1—C6—C7	120.64 (8)	C11—C12—N1	105.80 (8)
O1—C7—C6	112.55 (7)	C11—C12—H12A	127.1
O1—C7—C8	107.72 (7)	N1—C12—H12A	127.1
			
C6—C1—C2—C3	−1.16 (13)	O1—C7—C8—C9	52.81 (10)
C1—C2—C3—C4	−0.15 (14)	C6—C7—C8—C9	176.07 (7)
C2—C3—C4—C5	1.41 (14)	C10—N1—C9—C8	−107.01 (10)
C3—C4—C5—C6	−1.37 (14)	C12—N1—C9—C8	71.41 (11)
C4—C5—C6—C1	0.06 (13)	C7—C8—C9—N1	59.04 (10)
C4—C5—C6—C7	179.56 (8)	C11—N2—C10—N1	−0.15 (11)
C2—C1—C6—C5	1.20 (12)	C12—N1—C10—N2	0.31 (11)
C2—C1—C6—C7	−178.30 (8)	C9—N1—C10—N2	178.99 (8)
C5—C6—C7—O1	−129.62 (8)	C10—N2—C11—C12	−0.07 (11)
C1—C6—C7—O1	49.88 (10)	N2—C11—C12—N1	0.25 (11)
C5—C6—C7—C8	109.98 (9)	C10—N1—C12—C11	−0.33 (10)
C1—C6—C7—C8	−70.52 (10)	C9—N1—C12—C11	−179.01 (8)

### Hydrogen-bond geometry (Å, º)

Cg1 is the centroid of the C1–C6 phenyl ring.

**Table:**

*D*—H···*A*	*D*—H	H···*A*	*D*···*A*	*D*—H···*A*
O1—H1*O*1···N2^i^	0.964 (19)	1.89 (2)	2.8432 (12)	171.7 (17)
C1—H1*B*···O1^ii^	0.95	2.48	3.4188 (12)	172
C10—H10*A*···*Cg*1^iii^	0.95	2.68	3.4778 (12)	142
C12—H12*A*···*Cg*1^iv^	0.95	2.69	3.5373 (11)	149

Symmetry codes: (i) −*x*+1, *y*−1/2, −*z*+1/2; (ii) −*x*+1, −*y*, −*z*+1; (iii) −*x*+1, *y*+1/2, −*z*+1/2; (iv) −*x*, *y*+1/2, −*z*+1/2.
